# Developing stable, simplified, functional consortia from *Brachypodium* rhizosphere for microbial application in sustainable agriculture

**DOI:** 10.3389/fmicb.2024.1401794

**Published:** 2024-05-23

**Authors:** Mingfei Chen, Shwetha M. Acharya, Mon Oo Yee, Kristine Grace M. Cabugao, Romy Chakraborty

**Affiliations:** Department of Ecology, Earth and Environmental Sciences Area, Lawrence Berkeley National Laboratory, Berkeley, CA, United States

**Keywords:** rhizosphere microbiome, high-throughput enrichment, carbon substrates, slow and fast growing, reduced complexity consortia, network analysis

## Abstract

The rhizosphere microbiome plays a crucial role in supporting plant productivity and ecosystem functioning by regulating nutrient cycling, soil integrity, and carbon storage. However, deciphering the intricate interplay between microbial relationships within the rhizosphere is challenging due to the overwhelming taxonomic and functional diversity. Here we present our systematic design framework built on microbial colocalization and microbial interaction, toward successful assembly of multiple rhizosphere-derived Reduced Complexity Consortia (RCC). We enriched co-localized microbes from *Brachypodium* roots grown in field soil with carbon substrates mimicking *Brachypodium* root exudates, generating 768 enrichments. By transferring the enrichments every 3 or 7 days for 10 generations, we developed both fast and slow-growing reduced complexity microbial communities. Most carbon substrates led to highly stable RCC just after a few transfers. 16S rRNA gene amplicon analysis revealed distinct community compositions based on inoculum and carbon source, with complex carbon enriching slow growing yet functionally important soil taxa like Acidobacteria and Verrucomicrobia. Network analysis showed that microbial consortia, whether differentiated by growth rate (fast vs. slow) or by succession (across generations), had significantly different network centralities. Besides, the keystone taxa identified within these networks belong to genera with plant growth-promoting traits, underscoring their critical function in shaping rhizospheric microbiome networks. Furthermore, tested consortia demonstrated high stability and reproducibility, assuring successful revival from glycerol stocks for long-term viability and use. Our study represents a significant step toward developing a framework for assembling rhizosphere consortia based on microbial colocalization and interaction, with future implications for sustainable agriculture and environmental management.

## 1 Introduction

Rhizosphere microbes have co-evolved with host plants and usually form mutually beneficial relationships. In return for carbon in the form of root exudates (Bais et al., 2006; De Deyn et al., 2008; Hu et al., 2018; Vives-Peris et al., 2020), rhizosphere microbes perform a range of functions that benefit their host plants. These encompass the conversion of essential nutrients (e.g., nitrogen, phosphate, zinc, iron) into more accessible forms for plant assimilation (Tariq et al., 2007; Sharma et al., 2013; Kuan et al., 2016; Satyaprakash et al., 2017; Pérez-Izquierdo et al., 2019), conferring resilience against environmental stressors such as pathogen infections and water limitations (Rodriguez et al., 2008; Mendes et al., 2011; Bulgarelli et al., 2013; Bhattacharyya et al., 2021), secretion of plant growth promoting hormones (van der Lelie et al., 2009; Bulgarelli et al., 2013) among others. Given their high abundance, diversity and activity, rhizosphere microbes are often considered as “the second genome of plants” (Berendsen et al., 2012). To advance our understanding and gain more insight into rhizosphere assembly processes, it is critical to recover and to recreate a representative rhizosphere community that accurately encapsulates the inherent diversity and functions of the rhizobiome for detailed lab investigations.

Generally, representative reduced complexity consortia (RCC) are constructed employing bottom-up and top-down approaches. Bottom-up method focuses on building synthetic communities from cultivated isolates. However, this method could inadvertently exclude microbes that resist cultivation under routine cultivation and incubation conditions including some rare and dormant taxa (de Souza et al., 2020; McClure et al., 2020; Choi et al., 2021). In addition, for subsequent consortia stability, knowledge about individual microbes and their interactions with partner microbes is imperative (Kaeberlein et al., 2002; Gilmore et al., 2019; Wu et al., 2020). Conversely, the top-down approach employs enrichments to generate consortia of reduced complexity after multiple passages (Gilmore et al., 2019; Díaz-García et al., 2021). However, this approach may only yield fast-growing generalists of low diversity if routine carbon sources are used, overlooking the true rich phylogenetic and functional diversity of rhizosphere (Kaeberlein et al., 2002; Wu et al., 2020). Consequently, vital to construction of useful RCC is the design framework, that demonstrates stability, complexity, reproducibility, scalability, and genetic tractability—which appears to be generally lacking (Wu et al., 2020; Liang et al., 2022).

In this study, we developed a systematic and standardized framework to generate RCC that optimally represent the rhizosphere microbiome of *Brachypodium distachyon* and satisfies all the above criteria. Previously, we grew young *Brachypodium distachyon* using natural soil in standardized fabricated ecosystems (EcoFABs; Zengler et al., 2019) as well as conventional pots and tubes under controlled conditions, and demonstrated that the rhizosphere microbiome of *Brachypodium* was clearly distinct from bulk soil microbiome irrespective of the growth container (Acharya et al., 2023). Subsequently, in this current study, using the naturally selected co-localized root microbiome as inoculum, we performed a large number of high-throughput enrichments to derive rhizobiome relevant RCC. To do so, we considered critical parameters such as choice of carbon substrate, differential microbial growth rates and even root-enriched inoculum from different growth containers. We measured the diversity and richness of the developing communities across multiple generations, identified the key microbial taxa that were significantly influenced by these factors, and discerned the keystone taxa that controls the interactions within the core community members. Furthermore, we demonstrated the validity of our framework showing stability, tractability, reproducibility, and revivability from both original enrichments and preserved glycerol stocks, all key factors important for effective use and application of microbial consortia for field studies. Our approach of generating RCC using this robust and standardized framework that considers co-localization, and microbial interactions is a significant advance toward microbial-based solutions for agriculture and ecosystem management toward climate change mitigation strategies.

## 2 Materials and methods

### 2.1 Microbial community inocula

The enrichment inoculum consisted of tightly root-associated rhizosphere soil that came from *Brachypodium distachyon* grown in soil collected from Angelo Coast Range Reserve (pH 5.75 ± 0.37), California (39° 44′ 21.4′′ N 123° 37′ 51.0′′ W) and in three types of containers: fabricated ecosystems (EcoFABs), pots and test tubes (Acharya et al., 2023). The loosely bound and tightly bound soil from the base and tip of the 14-day old *Brachypodium distachyon* roots were combined from each container type in order to prepare inocula for subsequent enrichments. The rhizosphere microbes loosely attached to the roots was extracted by vortexing the roots in 5 mM sodium pyrophosphate for 15 s, repeated three times. Subsequently, the roots were immersed in fresh pyrophosphate buffer and sonicated for 5 min to release the tightly-bound microbes fraction. The extract was then diluted 100-fold and stained with 1 μL of SYTO9 and Propidium Iodide per mL. After incubating for 15mins in the dark, the sample was analyzed using a flow cytometer (AttuneNxT, ThermoFisher) to determine the total cell numbers from inocula of each container type was normalized to 4.6 × 10^6^ cells mL^−1^ in RCH2 media. 10% (v/v) of these resulting soil slurries (180 μL) was used to initiate inoculate enrichments.

### 2.2 Media for enrichment

The base media for all but one enrichment was a modified RCH2 media (minimal media) at pH 6.0 to mimic the field pH (Chakraborty et al., 2017; Supplementary Table S1). After autoclaving, we added 10 mL/L of Wolfe’s vitamins (Balch et al., 1979). Carbon source stocks were prepared in MilliQ water at 20 mM, filter (0.2 μm) sterilized, and added to sterile RCH2 media at a final concentration of 2 mM. The concentration is set to mimic the actual measured root exudates (~470 mg/L) from other grasses (Zhalnina et al., 2018). For carbon sources, single carbon sources dominant at *Brachypodium distachyon* root exudates were used: citrate, malate, glucose, asparagine, glutamine, glucuronic acid, and a mixture of all these carbon sources (also at 2 mM). Lastly, 1/10th dilution of commercially available media, R2A (hereafter referred to as 1/10 R2A) was used as an additional media for reference since R2A has been widely adopted for enrichment and isolation of soil and subsurface microbes (Jenkins et al., 2017; Zhalnina et al., 2018; Wu et al., 2020; de Raad et al., 2022; Gushgari-Doyle et al., 2022). However, in keeping with the carbon concentrations in the other enrichment media, it was diluted 10-fold to mimic similar carbon concentrations as other carbon sources (2 mM) and adjusted to pH 6. The average TOC concentration of the 1/10 R2A from triplicates was measured to be 105.8 mg/L, and the calculated TOC concentration of other carbon sources ranged from 264.24 to 384.248 mg/L (Supplementary Table S2). To note that composition of R2A media is known to slightly vary between manufactured batches.

### 2.3 High-throughput enrichment and sampling

High-throughput (HT) enrichments via sequential transfers in 96-well plates were performed to get reduced complexity communities (Supplementary Figure S1). Each well had 1.8 mL volume in total with 10% inoculum (180 μL) from *Brachypodium distachyon* rhizosphere samples as detailed above and medium with either 2 mM carbon source in RCH2 medium or 1/10 R2A. Initially, our experimental design involved two separate sets of deep-well plates, designated as Plates A and B. These plates were prepared to test a variety of conditions, including different carbon sources and original inocula sourced from three distinct containers, with four replicates per condition in one plate. It is important to emphasize that Plates A and B were not intended to serve as replicates of one another. Instead, they were set up to accommodate and independently assess the diverse experimental variables under study. Thereafter, plates were transferred 10 times at two different intervals, 3 days and 7 days for targeting fast and slow growing microbes. Each transfer is referred to as a “Generation,” which indicates how many transfers have occurred (e.g., Gen 6 means it is the 6th transfer of the original inoculum). Plates were incubated in the 30°C shaking incubator at 130 rpm, after sealing with a breath-easy seal (Parafilm). Every 3 or 7 days, 180 μL (10%) of culture was transferred to new plates with fresh media. Glycerol stocks (80 μL culture + 40 μL glycerol) were prepared at each transfer and frozen at −80°C. Remaining culture was pelleted down for DNA extraction and sequencing. Ultimately, 20 plates containing 1,920 samples were obtained during the enrichment process.

### 2.4 Validation of derived consortia

In order to evaluate our derived consortia, we first assessed microbial stability over time from our enrichment experiments. The volatility analysis was implemented as previously described (Halfvarson et al., 2017), in which the weighted UniFrac distance between samples with adjacent timepoints (e.g., Gen 1 vs. Gen 3) for any given subject was calculated from the “rbiom” package (Smith, 2023). In general, the lower values of calculated volatility equal to higher stability. We also compared the species richness of our derived consortia with the original source (soil and rhizosphere). Apart from assessing our enrichments, we further assessed the reproducibility and revivability of our derived microbial consortia stored in glycerol stocks. We selected enrichments from 1/10 R2A medium for their practicality for potential commercialization. We selected five glycerol stocks based on the criterion of including taxa that are less abundant but vital and challenging to culture. These stocks are from the 3-day transfers (3B9-H4, 3B9-H11) and the 7-day transfers (7B6-H1, 7B6-H8, and 7B6-H11). The naming convention (e.g., “3B9-H4”) indicates a 3-day or 7-day time interval, plate ID (A or B), generation 9 or 6, and followed by the specific location of a glycerol stock in a 96 deep well plate after the hyphen. Glycerol stocks were completely thawed (120 μL) and serially subcultured (10% v/v inoculum and 90% v/v 1/10 R2A media) at 3- or 7-day intervals to bring the final volume up to 100 mL. 80 mL of the final culture was then spun down as pellets for DNA extraction, 10 mL was used for the next round of subculture, and the remaining was saved as 10 glycerol stocks (1 mL culture +0.5 mL glycerol). The reproducibility of our consortia was tested by continuing subculturing using a 10% v/v inoculum every 3 or 7 days, totaling 5 transfers. For revivability tests, the preserved glycerol stocks from the first round of subculture were thawed and serially subcultured to bring up the volume to 100 mL, and 80 mL of the final culture was used for DNA extraction.

### 2.5 16S rRNA gene amplicon data processing and amplicon sequence variants analysis

Four timepoints (transfers 1, 3, 6, and 9) from enrichment experiments along with samples from stability tests were prepared for DNA extraction. Genomic DNA of these samples were extracted at UC Berkeley DNA Sequencing Facility and submitted to Novogene Corporation Inc. for 16S amplicon sequencing of the V4 region using the universal bacterial primers 515F (GTGCCAGCMGCCGCGGTAA) and 806R (GGACTACHVGGGTWTCTAAT). The method previously published (Wu et al., 2020) was employed to analyze the amplicon sequence variants (ASVs). In summary, analysis of microbial community sequences was conducted using QIIME 2020.8 (Bolyen et al., 2019). Novogene provided demultiplexed sequences that were imported using the Phred33V2 variant. Quality filtering and denoising steps were performed with DADA2 (Callahan et al., 2016) and amplicon sequence variants were aligned with MAFFT (Katoh et al., 2002). Taxonomy was assigned using the Silva reference database v138 from 515F/806R region of sequences classifier, using a cutoff at 99% for ASVs (Quast et al., 2013; Yilmaz et al., 2014; Bokulich et al., 2018; Robeson et al., 2020). The phylogenetic tree of enriched ASVs was constructed with RAxML (Stamatakis, 2014), and visualized using iTOL (Letunic and Bork, 2021).

### 2.6 Data analysis and statistics

Statistical analyses of the 16S datasets were conducted using R software using the “phyloseq” and “vegan” packages (Jari, 2007; McMurdie and Holmes, 2013). We created two distinct phyloseq objects for the analyses, focusing on (1) taxonomic analysis of the initial enrichments for consortia generation and (2) evaluation of consortia reconstituted from glycerol stocks. Each analysis involved generating separate phyloseq datasets, which were then normalized by rarefying to a consistent sequencing depth. The sequencing read depths used for each dataset were 85,445 for the original enrichment communities and 87,017 for the reconstituted glycerol samples. Potential contaminants in the samples were identified and removed using the R package “decontam” (Davis et al., 2018). Shannon’s diversity index (H′) and multivariate statistics were performed using the R package “vegan.” ASV distributions were transformed into relative abundances using the function “decostand.” These were subjected to Hellinger transformation before calculation of Bray–Curtis dissimilarity matrices comparing community composition between samples. Principal coordinate analysis (PCoA) using the function “vegdist” was performed using these dissimilarity matrices. A permutational multivariate analysis of variance (PERMANOVA) model was implemented in the vegan function “adonis” using Bray-Curtis distance matrix to evaluate the effect of carbon substrate, original inoculum, and transfer interval on community structure. The relative abundance of taxa among the samples were compared and enriched ASVs were selected using DESeq2 packages in R (Love et al., 2014).

We further used network analysis to understand the interspecies interactions from original enrichments and revived glycerol stock samples. We focused on 1/10 R2A samples from the original enrichments given their relatively higher diversity, and we also used 1/10 R2A as the growing media for the revived glycerol stocks. We identified core ASVs (present in at least 3 of the 4 generations for original enrichment, or present in at least 5 of the 6 generations for revived glycerol stocks experiments) and loaded their abundance matrix from all selected samples into R to construct and visualize the network using the “NetCoMi” package (Peschel et al., 2021). Besides, when comparing interspecies interactions of different generations, we also selected core ASVs using another threshold (present in at least 3 of the 4 replicates for 1/10 R2A enrichment samples for a given inoculum and transfer interval) and used “NetCoMi” package to generate networks. We generated a sparse matrix for network analysis using Pearson correlation with a threshold greater than 0.3 and conducted Student’s t-tests for Pearson correlation values with a significance cutoff at a *p*-value of less than 0.05. In this co-occurrence network, each node represents a single ASV. The edges connecting the nodes indicate a strong and significant correlation between the ASVs. The hub node (keystone taxa) is identified if the node’s eigenvector centrality value is above the 90% quantile. The clustering in this network was used to group the ASVs into modules that were densely connected within themselves but sparsely connected to other modules. The R script used in this manuscript can be found in https://github.com/mfchen2/RCC.

## 3 Results

### 3.1 Carbon sources and original inocula shape the diversity of enrichments

**Figure 1:**
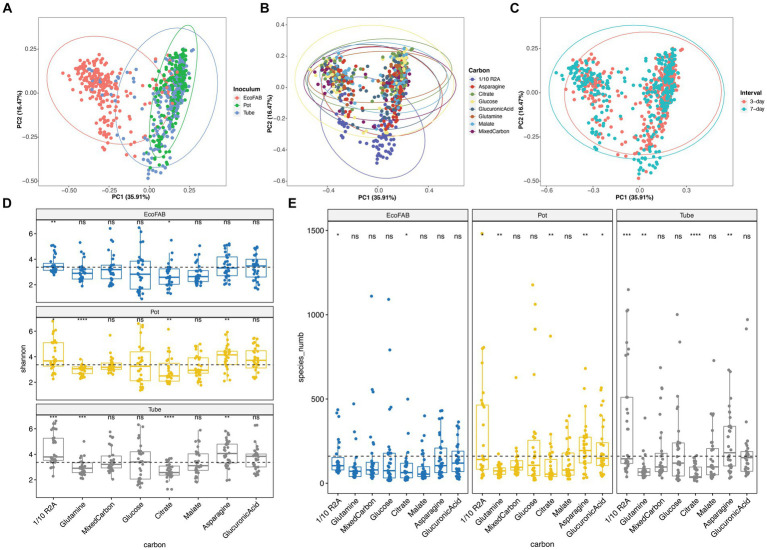
PCoA plot based on Bray-Curtis distance shows enrichment results comparing **(A)** original inoculum, **(B)** amended carbon substrates, and **(C)** transfer intervals. Boxplot showing **(D)** Shannon and **(E)** richness diversity indexes of enrichment results, separating by different containers and amended carbon substrates. For the boxplot, the dashed line in the plot indicates the global mean values for the measured diversity index for a given inoculum. Significant differences compared to global mean values of Shannon and richness indexes are indicated in asterisk (*). *, 0.05; **, 0.01; ***, 0.001; ****, <0.001. Analysis includes 768 enrichment samples.

All variables tested in the enrichment design (carbon substrate, inoculum from diverse growth containers, transfer interval) significantly influenced bacterial community structure in our enrichments from PERMANOVA results. Among them, original inoculum (*R*^2^ = 0.160, *p* < 0.001) and carbon sources (*R*^2^ = 0.149, *p* < 0.001) are important drivers of community dissimilarity, while sampling intervals (*R*^2^ = 0.030, *p* = 0.001) contributed to a lesser extent. We then visualized the grouped samples by PCoA ordination comparing different variables (Figures 1A–C), and used pairwise PERMANOVA comparisons to validate their statistical significance (Supplementary Table S3). When comparing across different inocula, microbial community from EcoFAB distinguished itself from pot or tube inocula (Figure 1A). Upon evaluating the impact of carbon substrates, significant variations in microbial community composition were observed for all (Supplementary Table S3), those amended with 1/10 R2A were markedly different from the rest (Figure 1B). Besides, pairwise PERMANOVA results also showed statistical differences for enrichments transferred at 3-day or 7-day intervals, although no distinct differences were observed solely based on observation of the PCoA plot (Figure 1C).

We also analyzed the impact of various tested variables on the biodiversity within enrichment cultures. Notably, enrichments originating from EcoFAB inocula displayed significantly lower Shannon diversity indices compared to those from pot and tubes (Supplementary Figure S2). With respect to carbon, regardless of inoculum source, citrate amendments resulted in notably lower biodiversity, whereas enrichments with 1/10 R2A medium exhibited significantly higher diversity (Figures 1D,E). Furthermore, asparagine and glutamine amendments were observed to induce significant shifts in the diversity index in pot and tube samples (Figures 1D,E). Finally, our findings reveal that the 3-day interval samples harbored significantly greater biodiversity than their 7-day counterparts (Supplementary Figure S2B).

### 3.2 Different carbon substrates can enrich unique rhizosphere taxa

**Figure 2:**
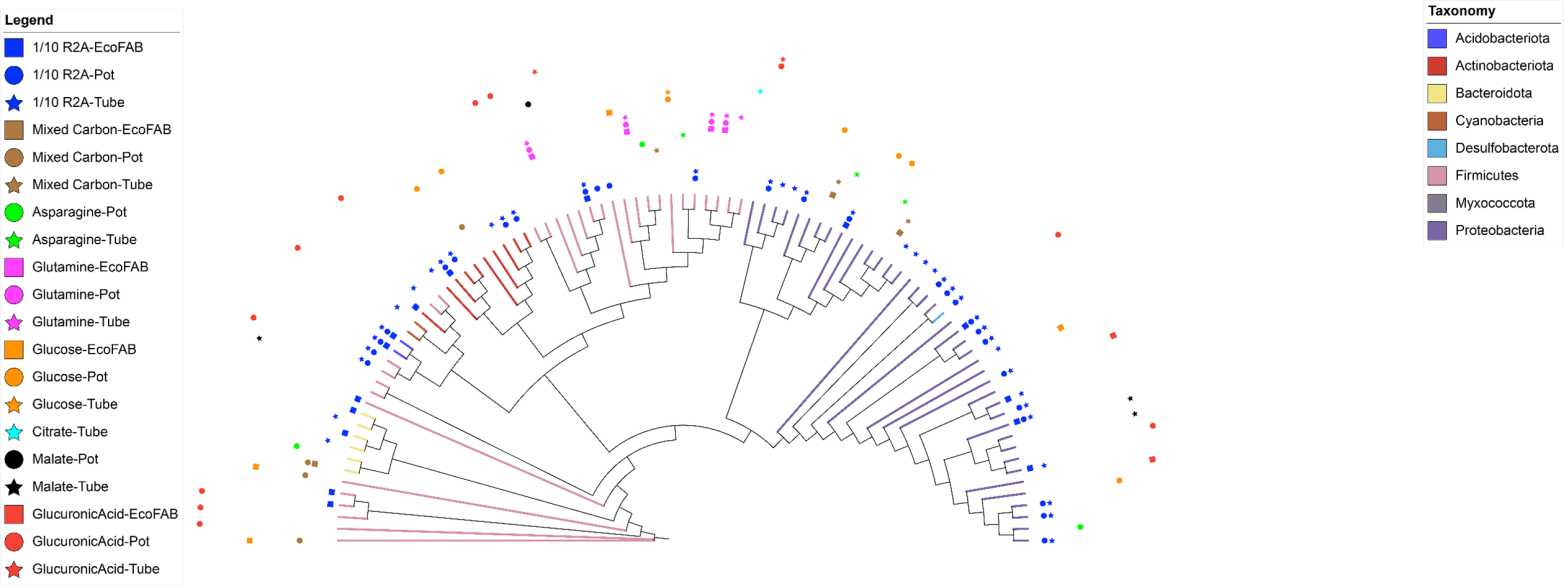
Neighbor joining tree of 93 ASVs which showed significant log fold changes during pairwise analysis of different carbon substrates. The ASVs were selected based on the following criteria: (1) ASVs significantly enriched in one carbon source compared to seven others from pairwise analysis (adjusted *p* < 0.05). (2) ASVs with a baseMean value greater than 5, and (3) ASVs occurring in only one sample are excluded. Different colors of the branches represent different phyla. Markers surrounding the tree denote the original inoculum (EcoFAB—square, Pot—circle, or Tube—star) and C substrate in which the ASV was significantly enriched. Analysis derives from 768 enrichment samples.

We quantitatively examined the impacts of carbon substrates on different taxa using differential gene relative abundance analysis (DESeq2). We first considered the impacts of different carbon on the differential enrichments of ASVs. To minimize the impact of the original inoculum in our analyses (Figure 1), we separated the samples based on inoculum and compared the ASVs of different carbon sources (Figure 2). We only include abundant taxa with a baseMean value > 5, and remove potential contaminants using the R package “decontam” (i.e., abundant ASVs that only present in one sample). This results in a total of 93 enriched ASVs. Generally, 1/10 R2A had the highest potential to enrich ASVs compared to other carbon sources. ASVs from genera *Terriglobus*, *Castellaniella*, *Arthrobacter*, *Luteibacter*, *Bacillus* and unclassified Rhizobiaceae (Supplementary Table S5) were significantly enriched in 1/10 R2A irrespective of inocula source. Glucose significantly enriched ASVs from genera *Delftia* and *Mycobacterium*, glucuronic acid significantly enriched ASVs from genus *Romboutsia*, and glutamine significantly enriched ASVs from genus *Brevibacillus* (Supplementary Table S5).

### 3.3 Complex carbon sources can enrich slow-growing but important rhizosphere microbes

**Figure 3:**
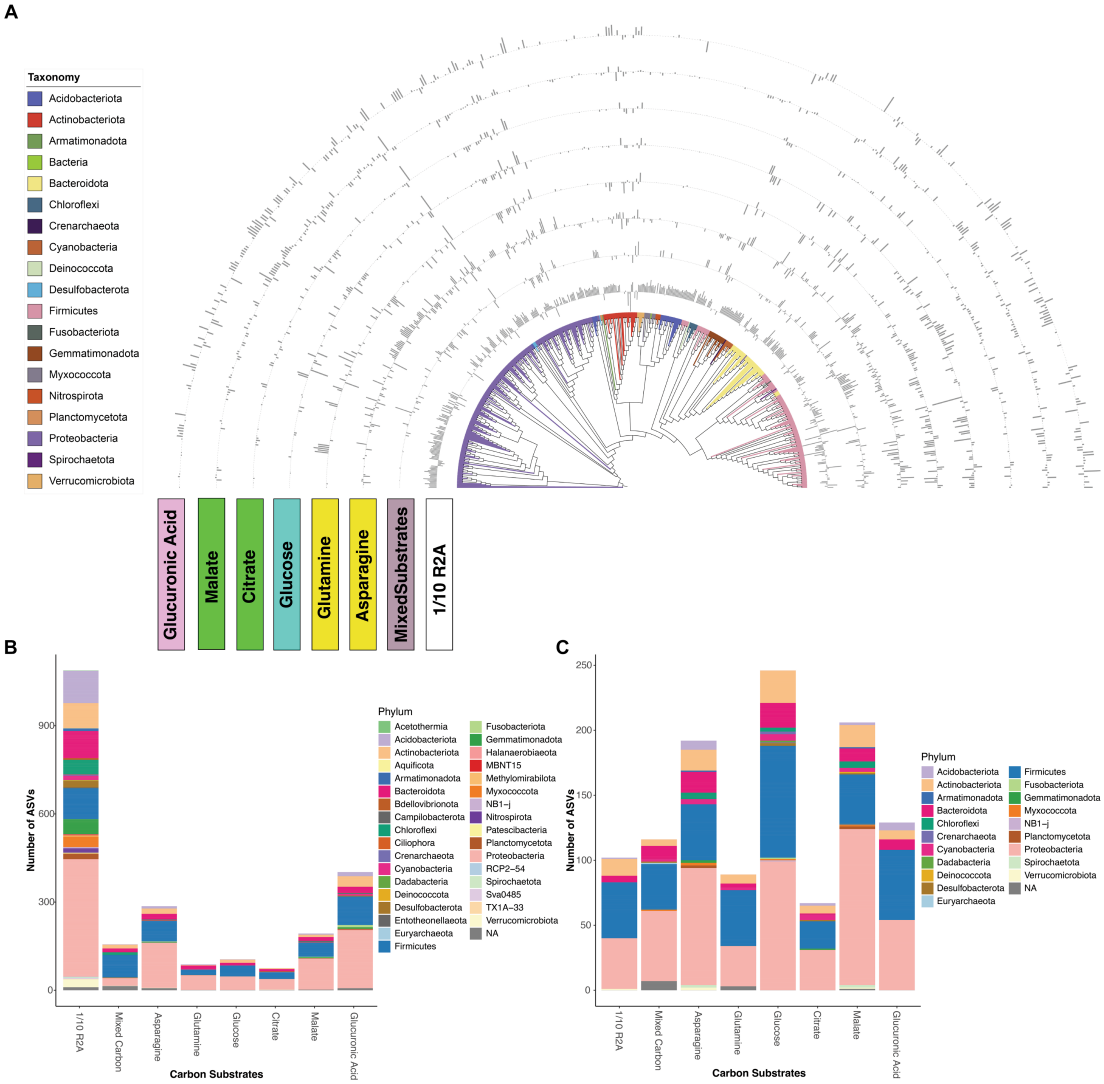
**(A)** Neighbor-joining tree of 530 differentially abundant ASVs showed significant log fold changes during pairwise analysis of transfer intervals (7-day or 3-day) from different carbon substrates. Bar charts around the tree represent log-fold changes for each ASV in each of carbon substrates (asparagine, glucose, glutamine, glucuronic acid, citrate, malate, mixed carbon, 1/10 R2A). An outward bar away from the tree represents a positive log fold change in the and an inward bar toward the tree represents a negative fold change in the respective ASV. Number of ASVs that are significantly enriched in **(B)** 7-day or **(C)** 3-day enrichment from different carbon substrates, different colors represent different phyla. Analysis derives from 768 enrichment samples.

Next, we compared how different carbon influenced microbes with different growth rates. In our experiments, we classified the growth rates of microbes using the following criteria: for a given carbon source and original inoculum, microbes significantly more abundant in 7-day interval samples compared to 3-day interval samples were categorized as “slow growers,” while the opposite were classified as “fast growers.” We only included Gen 6 and Gen 9 samples for DESeq2 analysis because Gen 6 and 9 represented later-stage transfers, primarily relying on amended carbon substrates as the principal carbon source, rather than the residual nutrients associated with the soil. Subsequent analyses revealed that 1/10 R2A medium, along with asparagine and glucuronic acid, demonstrated the greatest capability to enrich both slow and fast-growing microbes. In contrast, citrate and glutamine showed minimal enrichment potential for these microbial groups (Figures 3B,C). These results are consistent with Shannon diversity results comparing different carbon substrates (Figures 1D,E).

**Figure 4:**
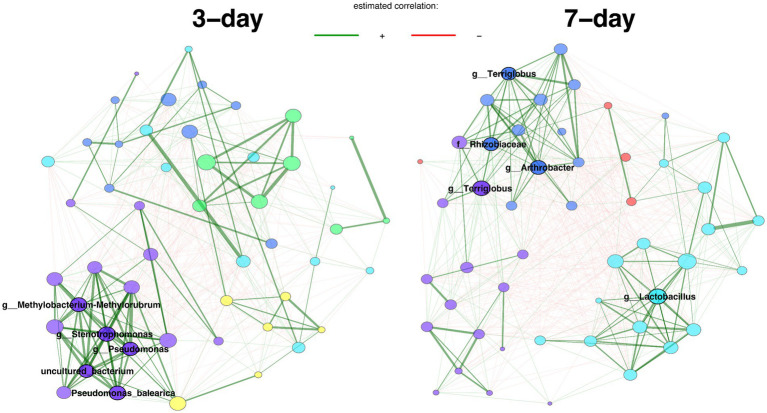
Comparison of bacterial associations between samples from 3-day and 7-day transfer intervals. Pearson correlation (>0.3) is used as an association measure, and t-test (< 0.05) is used as a sparse matrix generation method. Eigenvector centrality is used for defining hubs and scaling node sizes. Node colors represent clusters, which are determined using greedy modularity optimization. Clusters have the same color in both networks if they share at least two taxa. Green edges correspond to positive estimated associations and red edges to negative ones. The layout computed for the 3-day interval network is used in both networks. Hubs are highlighted with bold boundaries and corresponding taxon names. Analysis includes 96 enrichment samples.

We looked deeper into the more diverse 1/10 R2A enrichments, and used network analyses to identify the key taxa with different growth rates. 84 distinct core ASVs were identified for 3-day intervals and 91 distinct core ASVs for 7-day intervals. We used Jaccard indices to measure the similarity between the sets of most central nodes and hub nodes in the two centers, showing that two networks have significantly different network centralities (node, betweenness, closeness, eigenvector; Supplementary Table S4). Additionally, the keystone taxa (hubs) of the two networks were completely different. ASVs belong to genus *Methylobacterium-Methylorubrum*, *Stenotrophomonas*, *Pseudomonas* are hubs for 3-day interval transfers, while ASVs from family Rhizobiaceae, genus *Lactobacillus*, *Terriglobus*, and *Arthrobacter* are hubs for 7-day interval transfers (Figure 4).

### 3.4 Evaluation of the stability of constructed reduced complexity consortia

**Figure 5:**
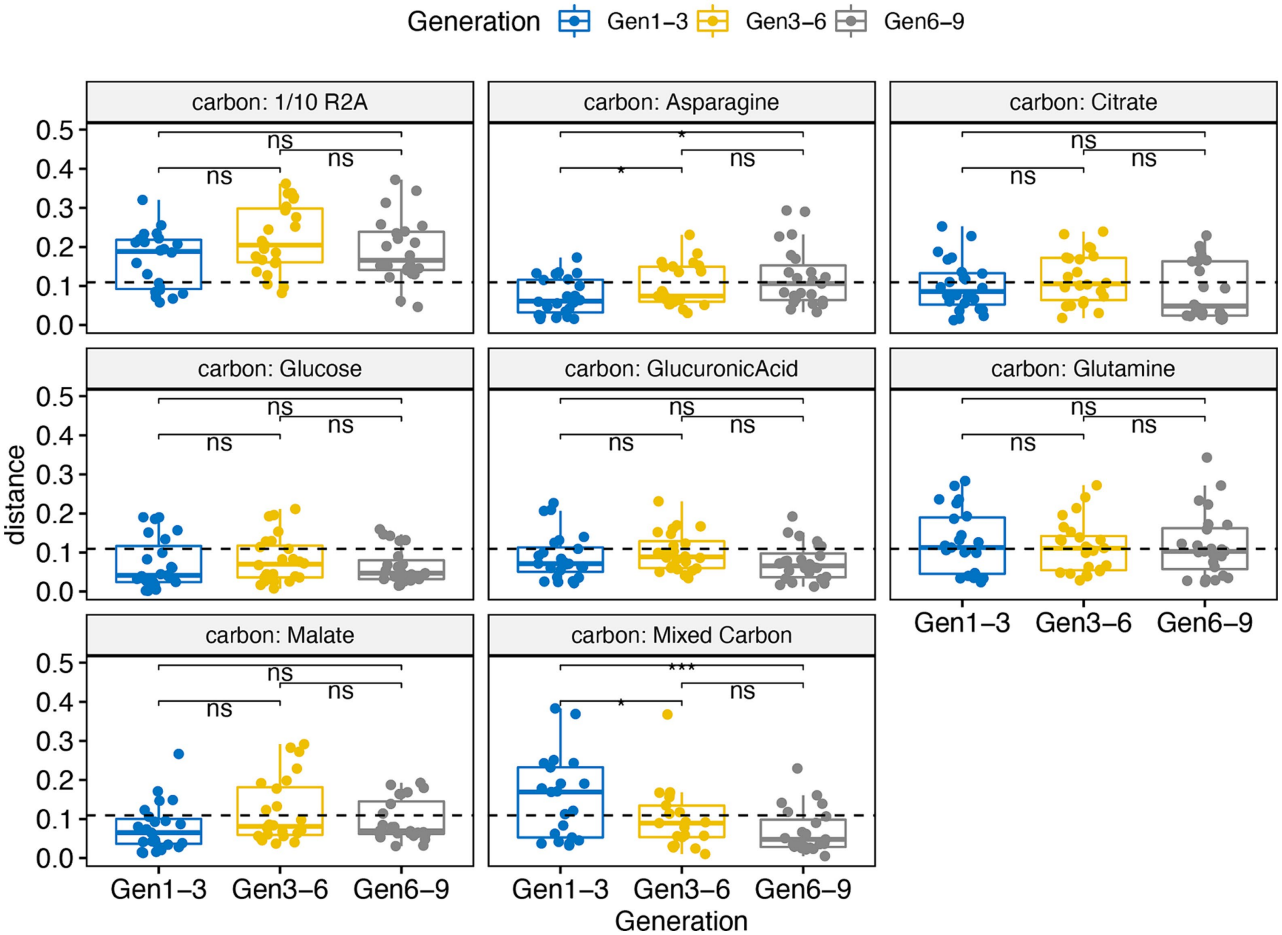
Volatility boxplot changes calculated from weighted Unifrac distance for samples amended with different carbon substrates comparing different generations. Gen 1–3 means differences between generation 1 and 3 which applies to other naming of x-axis. The dashed line is indicative of the global mean values of all calculated weighted Unifrac distances. Significance code: ***, 0.001; **, 0.01; *, 0.05; ns, not significant. Analysis includes 768 enrichment samples.

We assessed the effectiveness of our framework in constructing stable microbial consortia through a series of tests. First, we compared the species richness of our RCCs against original soil and rhizosphere samples, noting a significant reduction in species richness across all generations (Supplementary Figure S4). This reduction, observed irrespective of different container types, underscores the streamlined complexity of our RCC. Most treatments, with the exception of those amended with 1/10 R2A, maintained stable species richness across generations, with a majority of samples (457) exhibiting species richness below 70, indicating consistency in tractable species diversity (Supplementary Figure S5). Second, stability assessments showed that dominant taxa (genus > 1% in at least one sample) remained largely unchanged in relative abundance over time across various carbon sources, except for the 1/10 R2A amendments as mentioned above (Supplementary Figure S3). Moreover, analyses using weighted UniFrac distances between adjacent generations showed high stability across all carbon sources, except for 1/10 R2A. Mean distances for most carbon remained below the global average of 0.11, indicating their resilience over time (Figure 5). Third, network analyses of different adjacent generations (e.g., Gen 1 vs. Gen 3) revealed significant differences in centrality in some categories (Supplementary Table S9), yet adjacent generation networks exhibited similar clustering patterns, as indicated by the adjusted Rand index (Gen1-3: 0.228, Gen3-6: 0.148, Gen6-9: 0.114), with statistically significant *p*-values (*p*-values < 0.001). Notably, certain keystone species, including members of the Rhizobiaceae family and the genera *Paenibacillus*, *Sphingomonas*, *Mucilaginibacter*, and *Terriglobus*, were consistent across multiple generations, highlighting their pivotal roles within the consortia (Supplementary Figure S9).

**Figure 6:**
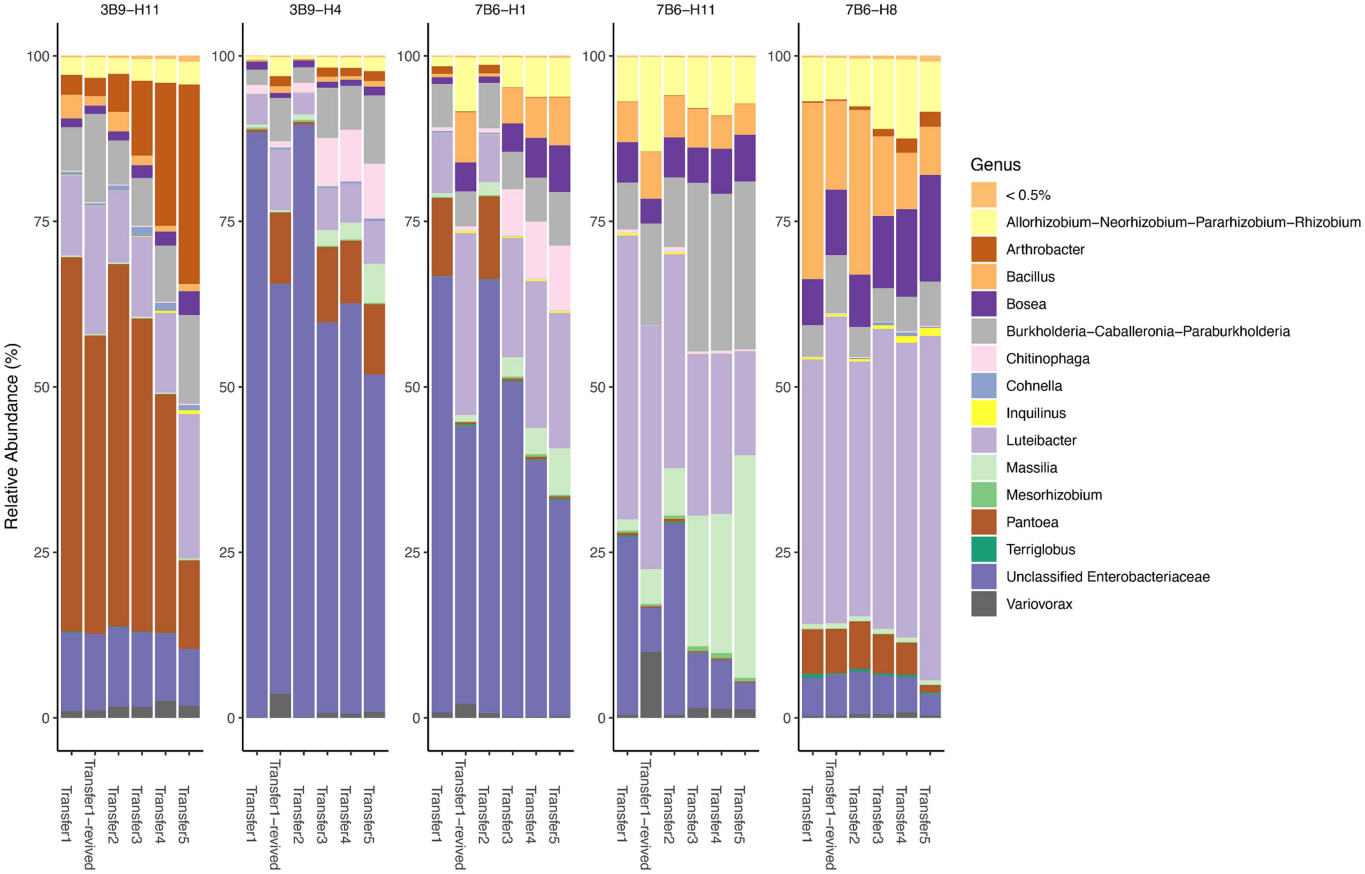
Temporal community structures of stability test of derived Reduced Complexity Consortia, reported as relative abundance of taxonomic genera (>0.5% relative abundance in at least one of the sample) from five different samples and five different transfers (including revived glycerol stock samples from one of the transfer). Analysis includes 25 samples from stability tests.

In a pivotal final step, we tested a smaller subset of five RCC that had been preserved in glycerol stocks for a few months to confirm their revivability, reproducibility, stability. These five glycerol stocks (selected from 3-day and 7-day transfers), encompass lower abundance taxa that play a critical role and present considerable culturing challenges. We serially transferred five times and compared the microbial community composition of each transfer. For stability, the dominant genera from the glycerol stocks (>0.5% in at least one sample) and their relative abundances remained consistent between transfers (Figure 6). Notably, some difficult-to-cultivate genera, such as *Terriglobus*, had originally low but very consistent relative abundances (0.1–0.5%) across different transfers. This observation aligns with prior research suggesting the survival and abundance of oligotrophic Acidobacteria during freeze–thaw events is significantly influenced by symbiotic relationships with other species, particularly select members of Proteobacteria (Juan et al., 2018). Besides, volatility tests showed that the mean weighted Unifrac distance of different transfers within samples was 0.042, and many of these (17 out of 25) had a distance < 0.05 (Supplementary Figure S7). Regarding reproducibility, richness and evenness of different transfers from the same starting inocula showed minimal changes across different generations (Shannon index change < 0.25 and species richness change < 10, Supplementary Figure S6). For revivability, the revived samples also show a similar microbial composition to the original subculture shown by low volatility (high stability; Supplementary Figure S6). We also performed network analysis on select revived RCC and identified 26 unique core ASVs across both 3-day and 7-day intervals. Contrary to prior network analysis findings, there were no significant differences between the central nodes of the two networks, as suggested by a small probability P (J ≤ j; Supplementary Table S7). The adjusted Rand index also presents a high similarity between the two clustering patterns (0.531, *p-*value = 0). Genus *Burkholderia* and *Mesorhizobium* were shared keystone taxa among 3-day and 7-day intervals, while family Enterobacteriaceae was annotated as keystone taxa for 3-day interval, and genus *Massilia* was annotated as keystone taxa for 7-day interval.

## 4 Discussion

A key innovation of our approach to enrichment of reduced complexity consortia from the rhizosphere is the multiplexed design of crucial parameters. This includes selection of naturally co-localized root microbes as inocula, selecting root exudate-mimicking carbon substrates, and implementing transfer intervals to allow both slow and fast-growing microbes. Moreover, we included *Brachypodium distachyon* grown in various containers to account for the potential impact of different growth chambers on rhizobiome interactions (Yee et al., 2021). Generally, microbial communities from EcoFAB were distinct from those sourced from pots and tubes (Supplementary Figure S2). This disparity may stem from inherent differences between EcoFAB and other containers due to space footprint or humidity control. These variations could directly influence root architecture (Yee et al., 2021) and root exudate composition and patterns, ultimately impacting the rhizobiome. Besides, previous study demonstrated the significant effect of inoculum on consortium richness (Zegeye et al., 2019). Our study further emphasizes the importance of inoculum composition as a critical factor when constructing reduced complexity microbial consortia.

Different root exudate compounds specifically favored the growth of certain ASVs, particularly those known for promoting plant growth. Asparagine, the dominant root exudate from *Brachypodium distachyon* (Kawasaki et al., 2016), significantly enriched ASVs from genera *Bradyrhizobium* and *Allorhizobium-Neorhizobium-Pararhizobium-Rhizobium* (Supplementary Table S5). Both *Bradyrhizobium* and *Allorhizobium-Neorhizobium-Pararhizobium-Rhizobium* are known to be associated with root nodules promoting plant growth (Mangeot-Peter et al., 2020; Jaiswal et al., 2021). *Mycobacterium* and *Delftia* were significantly enriched with glucose, both genera have been known to promote plant growth by solubilizing phosphate or fix nitrogen as non-rhizobial endophytes (Hakim et al., 2021; Zhou et al., 2021). *Brevibacillus*, significantly enriched in glutamine-amended samples, have been known previously to promote plant growth by inhibiting pathogenic microbes (Sheng et al., 2020). Interestingly, glucuronic acid significantly increased the diversity of ASVs enriched when inocula was derived from pots and enriched the second-highest number of distinct ASVs regarding different carbon substrates (Figures 1, 2). Although the general impact of glucuronic acids on the rhizosphere is relatively unexplored, recent studies indicate that glucuronic acid positively correlates with rhizosphere bacteria and is considered a key metabolite (Baker et al., 2022). Taken together, these results support that plants secrete diverse compounds through root exudates to selectively enrich for microbes with specific plant growth promoting traits. Therefore, our findings emphasize the importance of using relevant carbon substrates when attempting to enrich microbial consortia of varied phylogenies and functions from rhizosphere.

Moreover, choice of carbon substrates can distinctly influence the growth of fast- vs. slow-growing microbes. While the rhizobiome is typically believed to be dominated by fast-growing copiotrophs (Ling et al., 2022), slow-growing oligotrophs still play crucial roles including protection against plant diseases (van der Voort et al., 2016) and combating environmental stress (Hartman et al., 2018). Additionally, many slow-growing microbes are less abundant in the rhizosphere, making direct isolation and characterization challenging without intelligent enrichment efforts (Delmont et al., 2015). Our results show that diverse carbon substrates enriched similar fast-growing microbes (Figure 3C). This could be attributed to the fact that the original inocula originating from immature roots, are often dominated by fast-growing microbes (De Leij et al., 1995; Nacamulli et al., 2006). To note, glucose and malate, which have relatively simple chemical structures, can significantly enrich more of fast-growing microbes compared to slow-growing microbes (Figures 3B,C). However, distinct differences are observed in the enrichment potential of various carbon substrates for slow-growing microbes (Figure 3B). Notably, 1/10 R2A substantially enriched slow-growing microbes, including the phyla Acidobacteria and Verrucomicrobia, which are often recognized as difficult to enrich microbes (Sangwan et al., 2005; Ward et al., 2009) but with high potential for promoting plant growth (Kielak et al., 2016; Bünger et al., 2020). Interestingly, another complex carbon source (mixed carbon) does not significantly enrich slow-growing microbes as one might expect (Figures 3A,B). We think this observation could be attributed to several factors. First, R2A medium and its diluted version contain a broader range of metabolites compared to our mixed carbon substrates (de Raad et al., 2022), which could be crucial for enriching slow-growers, given their reliance on specific substrates for survival in the rhizosphere (Zhalnina et al., 2018). Second, the total organic carbon (TOC) concentration in 1/10 R2A is lower than in mixed carbon enrichment (Supplementary Table S2), potentially favoring oligotrophs that thrive in nutrient-limited environments. Third, we utilized consistent concentrations of different root exudates for our experiments, while in natural settings, these concentrations could vary substantially (Kawasaki et al., 2016), which might directly impact the enrichment of diverse microbes. These observations further underscore the critical role of selecting diverse and complex carbon sources to effectively enrich rare, yet essential, slow-growing microbes within the rhizosphere (Wu et al., 2020).

Additionally, fast- and slow-growing consortia showed stark differences in the keystone taxa (Figure 4), which are crucial nodes that hold central positions based on highest eigenvector centrality (Peschel et al., 2021). A node with high eigenvector means it connects to numerous central nodes, making it the influential node in the network analysis (Zhang, 2009). Consequently, keystone taxa can be considered ecologically important microbes responsible for shaping the microbial community structure and dynamics (Agler et al., 2016). Notably, 8 keystone taxa from the hubs belong to genera known to exhibit PGP traits (Kielak et al., 2016; Alijani et al., 2020; Jaiswal et al., 2021; Sah et al., 2021; Juma et al., 2022; Platamone et al., 2023). The robust link between keystone taxa and PGP traits underscores the significant impact of PGP bacteria on the dynamics of the rhizosphere microbiome (Pii et al., 2015), stressing the need for careful selection in constructing microbial consortia. Despite most keystone taxa displaying low (<0.5%) abundance across various generations under consistent conditions, they are persistent through different generations (Supplementary Table S8). Notably, specific keystone taxa, such as the Rhizobiaceae family and *Terriglobus* genus, remain constant across comparisons between fast and slow growers (Figure 4), as well as across different generations (Supplementary Figure S9). This underscores the vital role and involvement of keystone species, especially slow-growing microbes such as genus *Terriglobus*, in influencing rhizosphere dynamics, highlighting the necessity of incorporating such microbes into rhizosphere microbial consortia design and construction.

We evaluated the efficacy of our pipeline by assessing the stability and diversity of our enrichments over multiple generations (Supplementary Figure S1). We tested these two parameters because stability and tractability are two essential features for creating successful microbial consortia (Zegeye et al., 2019; McClure et al., 2020; Shayanthan et al., 2022). We found a significant reduction in species richness in our RCC-enriched samples across all generations (Supplementary Figure S4) from original inocula. Stability assessments across generations (1, 3, 6, and 9) demonstrated consistent low diversity (<70 observed species), and high consistency in species composition, regardless of the variables tested, highlighting the enriched samples’ stability (Figure 5; Supplementary Figure S5). This suggests that our reduced-complexity microbial communities likely achieved stability at early generations, except for those amended with 1/10 R2A medium, which showed increased diversity. Nonetheless, network analysis of 1/10 R2A samples across generations revealed consistent clustering (*p-*values < 0.001), indicating similar community structures despite the diversity increase (as shown in Supplementary Figure S9). These results affirm the effectiveness of our framework in developing stable and traceable microbial consortia.

As a last but critical step, we tested a subset of 5 of our microbial consortia that had been preserved in glycerol stocks to further assess their reproducibility, stability, and revivability after several months. Consistent revival and propagation from glycerol stocks is also a highly desired trait for microbial consortia to enable study across multiple labs and timescales and eventually for agricultural applications. Generally, our RCC exhibited consistent genera (in respect to both presence/absence and relative abundances, Figure 6), richness, and evenness across different transfers after revival (Supplementary Figure S6) and have tractable numbers (18 to 44) of observed OTUs, demonstrating good reproducibility. The low values from the volatility tests between transfers (Supplementary Figure S7) also support the stability of the derived microbial consortia. We also verified the revivability of our derived consortia, showing that the communities revived from glycerol stocks have a similar microbial composition to the original culture before freezing (Supplementary Table S6). Even though the glycerol stocks contained some dead cells, we upscaled the volume to 100 mL by serial transfers and subsequently centrifuged 80 mL of this to extract DNA. This process ensures that the sequencing results predominantly reflect the DNA from viable cells. Furthermore, our flow cytometry evaluation of the initial glycerol stocks and their serial transfers consistently showed a stable and substantial number of active cells (Supplementary Table S10). Network analysis revealed consistent patterns over different transfer periods and shared key taxa, including genera known for PGP traits (Nacamulli et al., 2006; Menéndez et al., 2020). Yet, it should be noted that each individual node may display varying likelihoods of interaction with other members within the same community when varying time intervals (Supplementary Figure S8). For instance, a node from the genus *Terriglobus*, which is known for slow growth rates (Kalam et al., 2020), demonstrates more active interaction with other nodes over 7-day intervals as denoted by the thickness of the edges. On the other hand, nodes from the *Cohnella* and *Chitinophaga* genera, recognized as fast-growing microbes (McKee and Brumer, 2015; Johnson et al., 2021), exhibited increased interactions with other nodes over 3-day intervals. These observations underscore the importance of longer incubation times when attempting to construct consortia, to ensure the inclusion of more slow-growing microbial members.

In summary, we developed a systematic high-throughput schema to enrich co-located microbes from the rhizosphere of *Brachypodium distachyon*, encompassing key factors like root exudates, inoculum source, and transfer duration. Our findings underscore the impact of the initial inoculum and carbon sources in shaping microbial community composition. We further highlight the influence of plant growth container type on enrichment outcomes, and our results demonstrate that diverse carbon sources similar to root exudates enhance specific beneficial microbes. We discovered that keystone taxa varied between fast- and slow-growing enriched communities and across different generations, however, they belong to microbes with plant-growth-promoting traits. Overall, our study represents a significant stride toward developing a framework for assembling stable, rhizosphere consortia based on microbial colocalization and interaction for agricultural and environmental enhancements.

## Data availability statement

The datasets presented in this study can be found in the NCBI BioProject repository: https://www.ncbi.nlm.nih.gov/bioproject/PRJNA990818.

## Author contributions

MC: Visualization, Validation, Methodology, Data curation, Conceptualization, Writing – review & editing, Writing – original draft. SA: Methodology, Data curation, Conceptualization, Writing – review & editing. MY: Methodology, Investigation, Conceptualization, Writing – review & editing. KC: Investigation, Conceptualization, Writing – review & editing. RC: Supervision, Resources, Funding acquisition, Conceptualization, Writing – review & editing, Writing – original draft.
