# Supplementary material for: Developing stable, simplified, functional consortia from Brachypodium rhizosphere for microbial application in sustainable agriculture
Source: Front Microbiol. 2024 May 23;15:1401794. doi: 10.3389/fmicb.2024.1401794 (PMC11153752; doi:10.3389/fmicb.2024.1401794)
Supplement: Supplementary file 1 [file Table_1.DOCX]

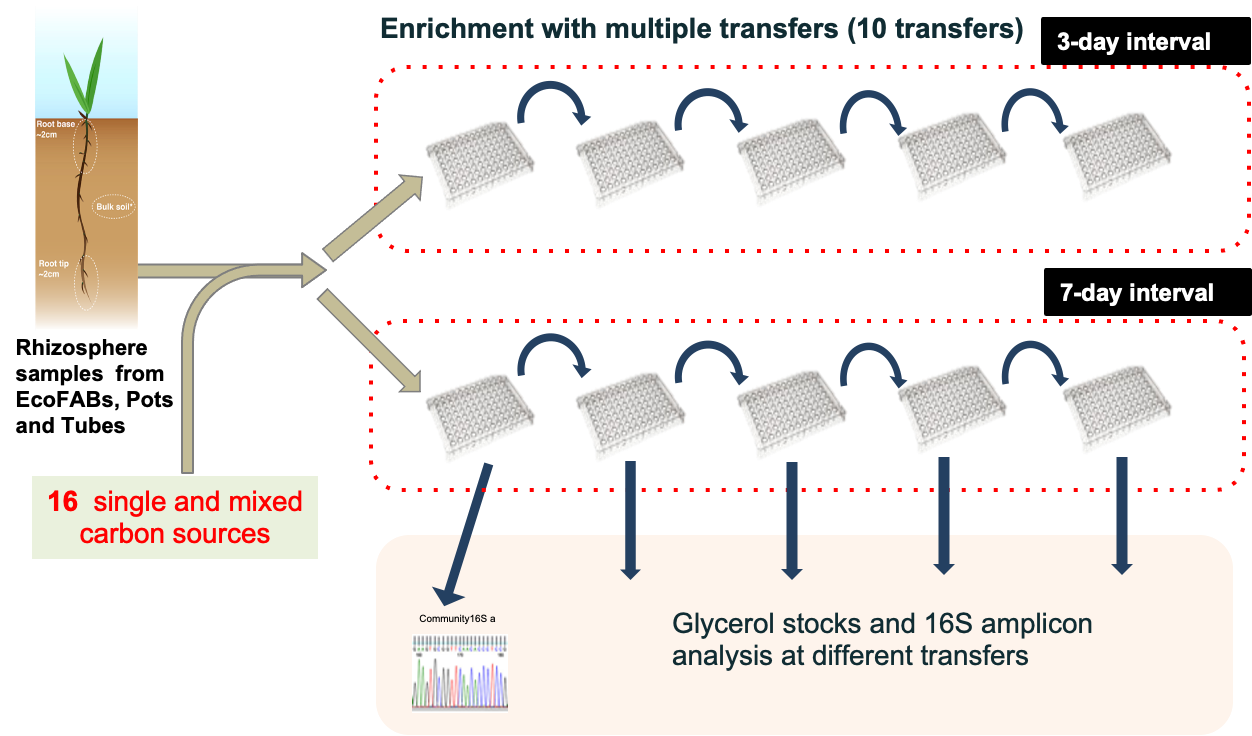


Supplementary Figure S1: Experimental design of the derived reduced complexity consortia pipeline


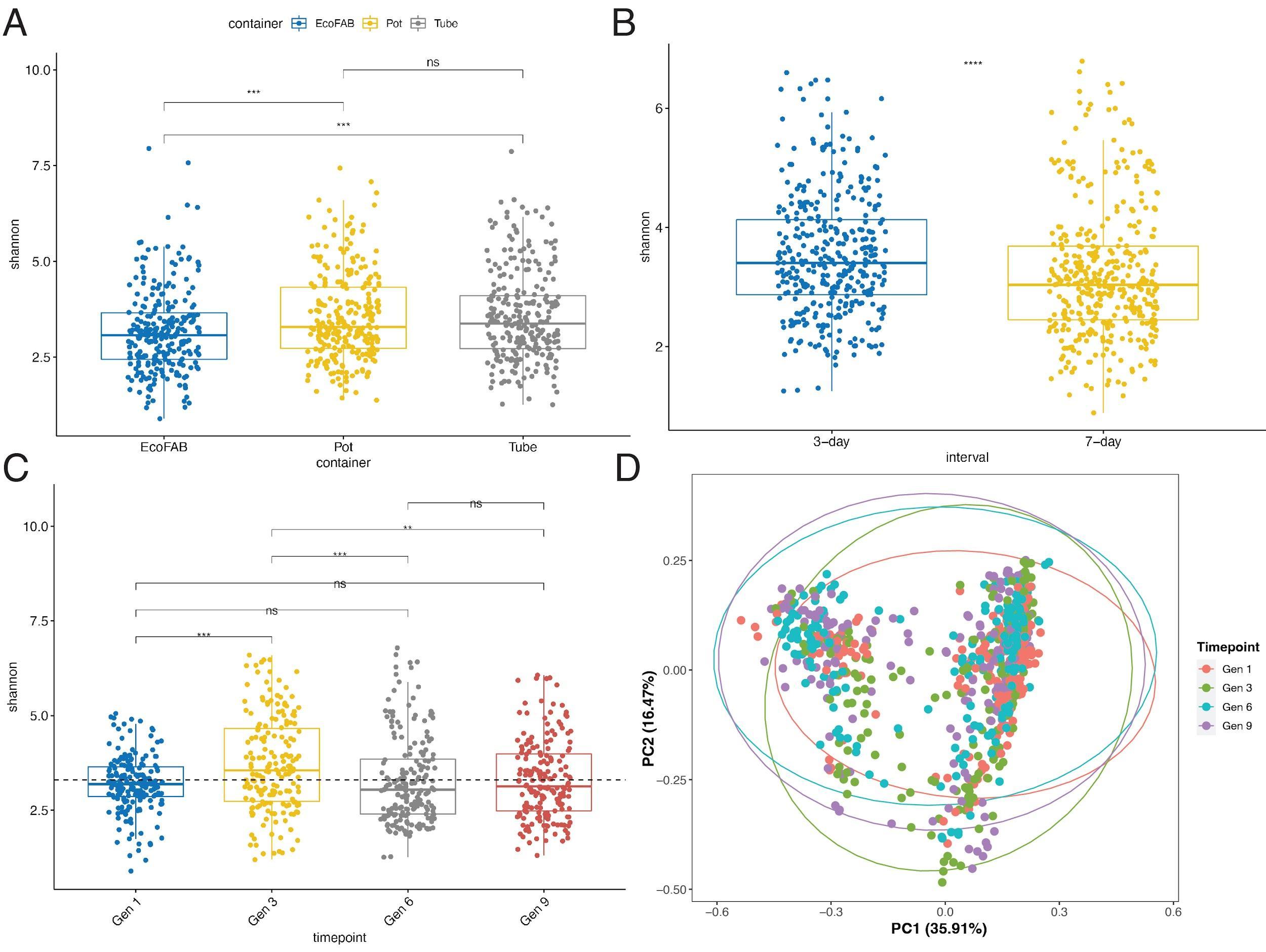


Supplementary Figure S2: Shannon Diversity comparing (A) original inoculum, (B) time intervals, (C) sampling generations, and (D) PCoA plot for sampling generations.

1. 1/10 R2A, 3-day
2. 1/10 R2A, 7-day

1. Mixed Carbon, 3-day

1. Mixed Carbon, 7-day

1. Malate, 3-day

1. Malate, 7-day

1. Glutamine, 3-day

1. Glutamine, 7-day

1. Glucuronic Acid, 3-day

1. Glucuronic Acid, 7-day

1. Glucose, 3-day

1. Glucose, 7-day

1. Citrate, 3-day

1. Citrate, 7-day

1. Asparagine, 3-day

1. Asparagine, 7-day

Supplementary Figure S3: Temporal community structures of different C-amended enrichments reported as relative abundance of taxonomic genera (> 1% relative abundance in at least one of the sample) from three different original inocula (EcoFAB, pot, tube), different generations and different time intervals.


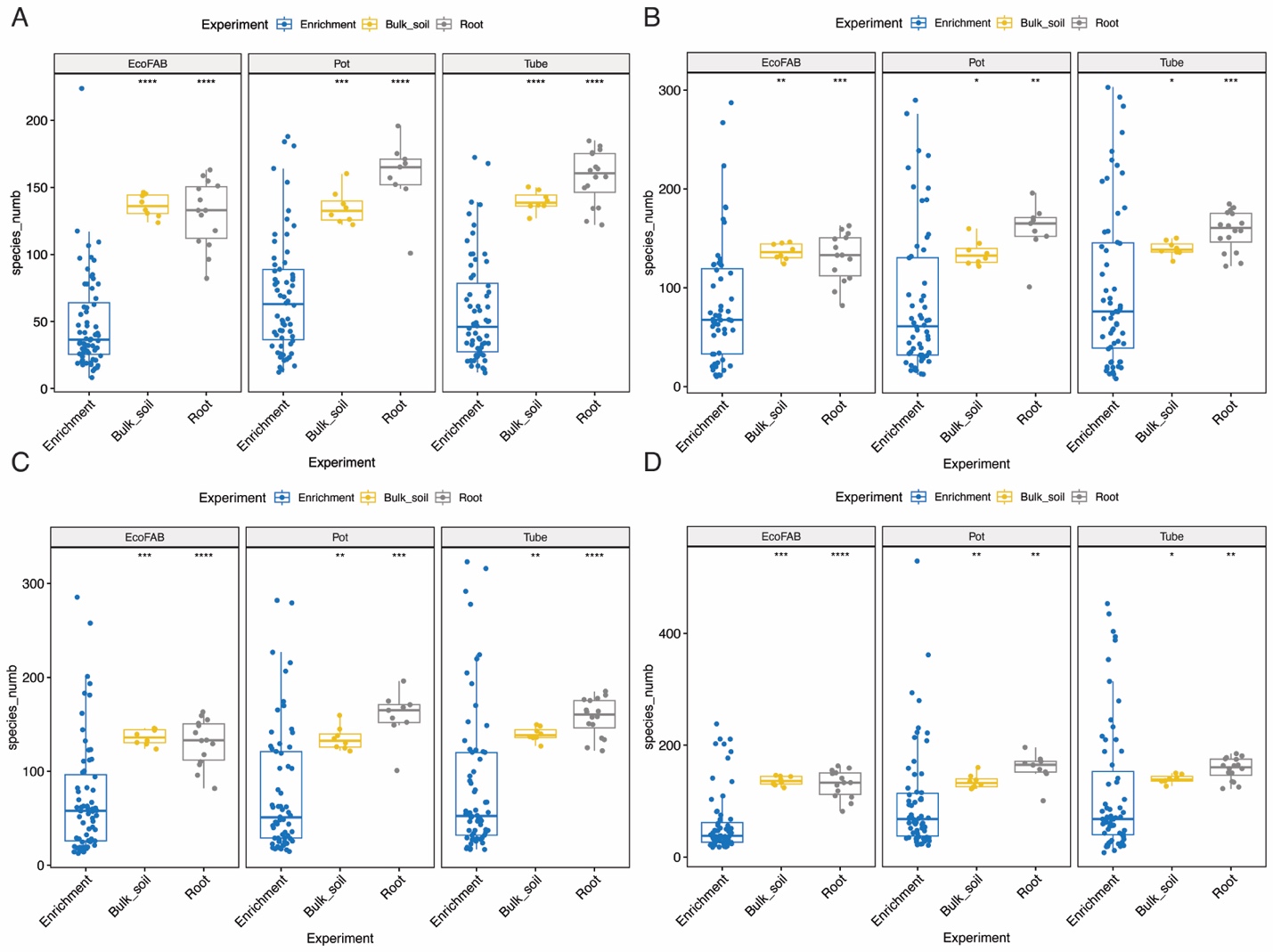


Supplementary Figure S4: Boxplot comparisons of species numbers (agglomerate to genus level) from biogeography and enrichment experiments of different generations (A: Gen1, B: Gen3, C: Gen6, D: Gen9). Wilcoxon-test p-values compared to the enrichment samples are indicated in asterisk (*). * = 0.05, ** = 0.01, *** = 0.001, **** = <0.001. The root tip/base samples and the bulk soil from biogeography experiments are included in this plot.


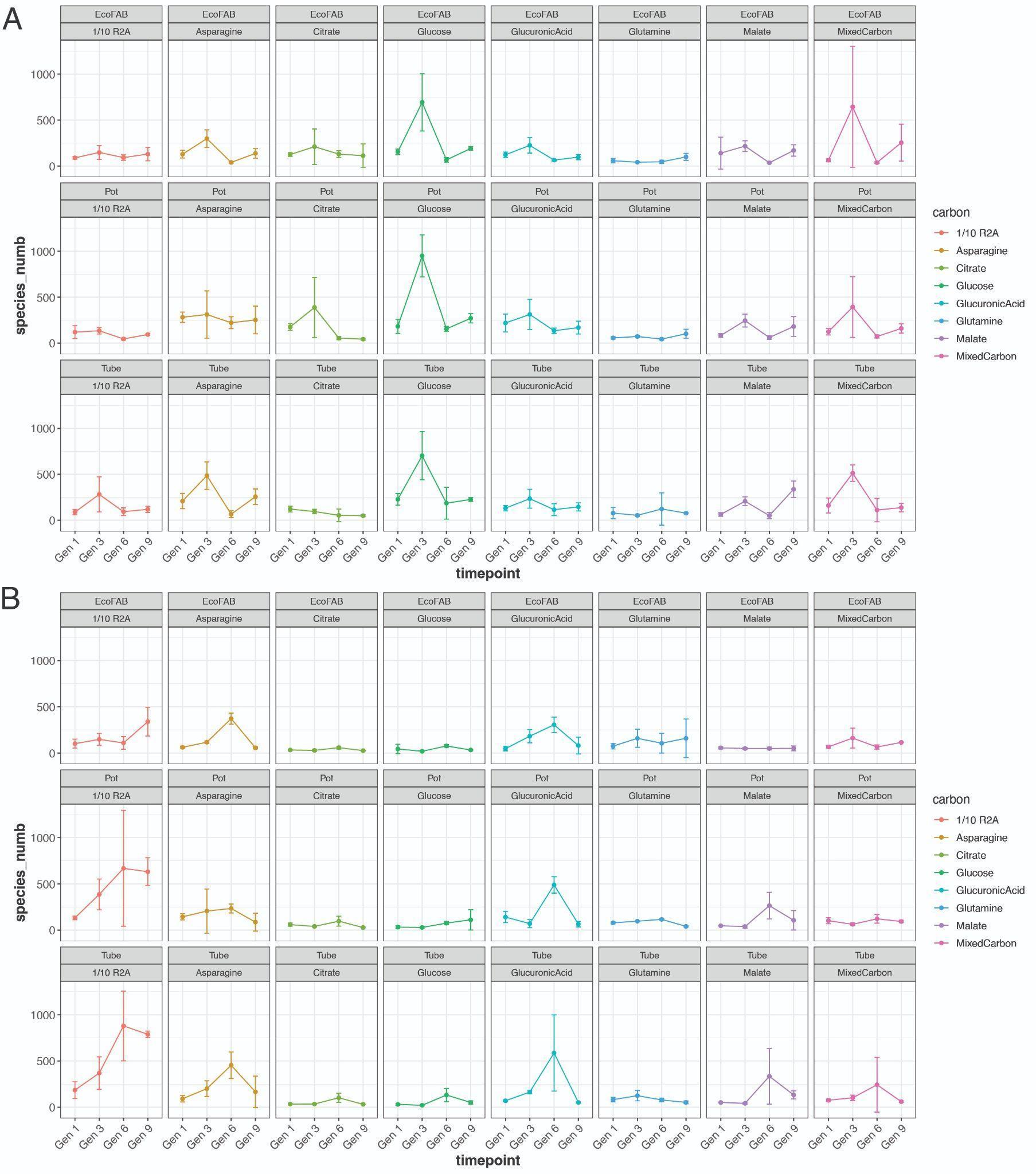


Supplementary Figure S5: Species richness boxplots for samples with different original inoculum and amended with different carbon substrates comparing different generations of (A) 3-day and (B) 7-day intervals. Species richness numbers are depicted by dashed lines from all samples using the same original inoculum.


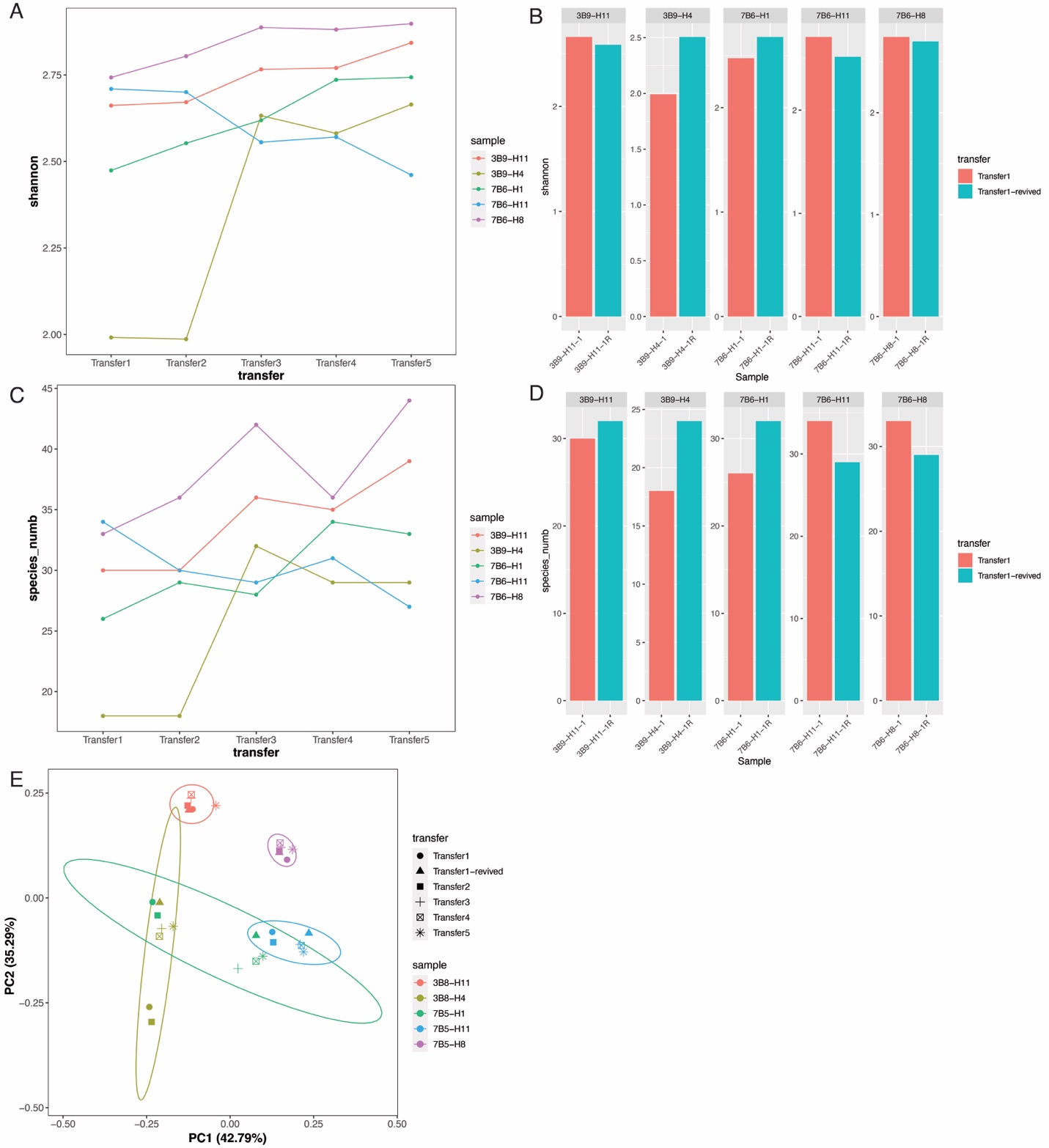


Supplementary Figure S6: Shannon index of (A) Line plots of samples of different transfers and (B) Barplots of transfer 1 and transfer 1 revived samples; Species number of (C) Line plots of samples of different transfers and (D) Barplots of transfer 1 and transfer 1 revived samples; (E) PCoA plots showing the comparisons of ASVs from consortia derived from different samples and different transfers.


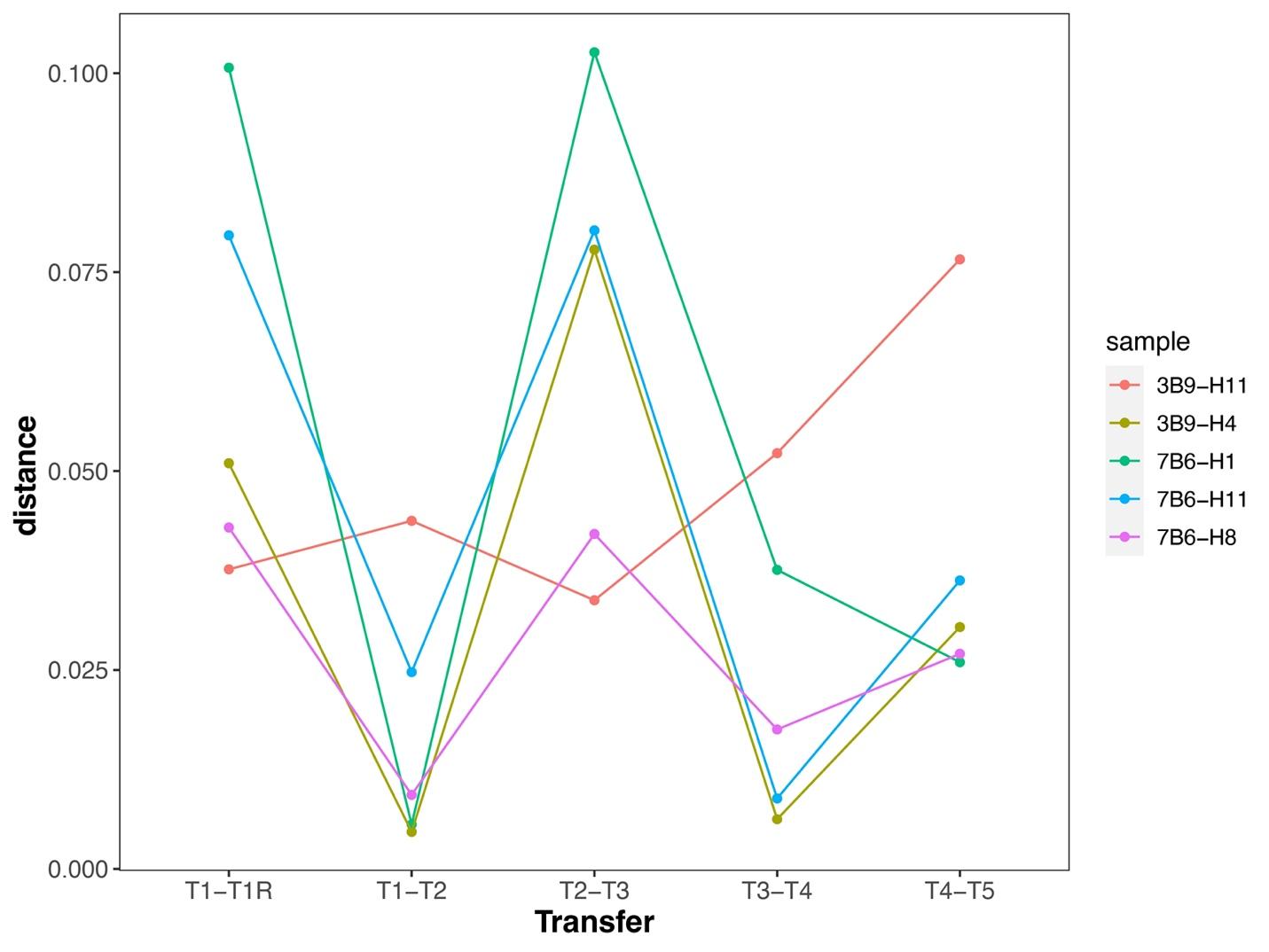


Supplementary Figure S7: Unifrac distances for samples between different transfers.


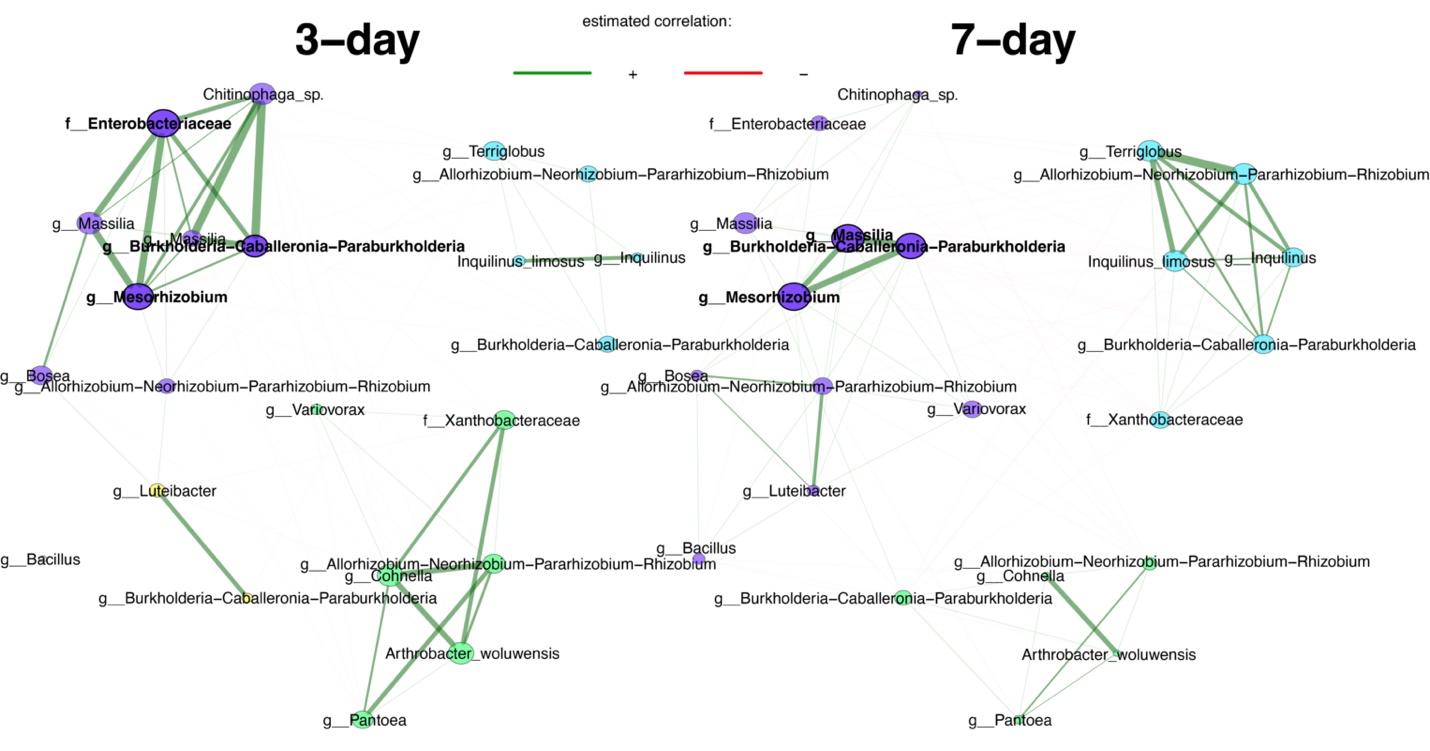


Supplementary Figure S8: Comparison of bacterial associations between regrowing consortia from glycerol stocks from 3-day and 7-day transfer intervals. Pearson correlation (>0.3) is used as an association measure, and t-test (< 0.05) is used as a sparse matrix generation method. Eigenvector centrality is used for defining hubs and scaling node sizes. Node colors represent clusters, which are determined using greedy modularity optimization. Clusters have the same color in both networks if they share at least two taxa. Green edges correspond to positive estimated associations and red edges to negative ones. The layout computed for the 3-day interval network is used in both networks. Hubs are highlighted with bold boundaries and corresponding taxon names.


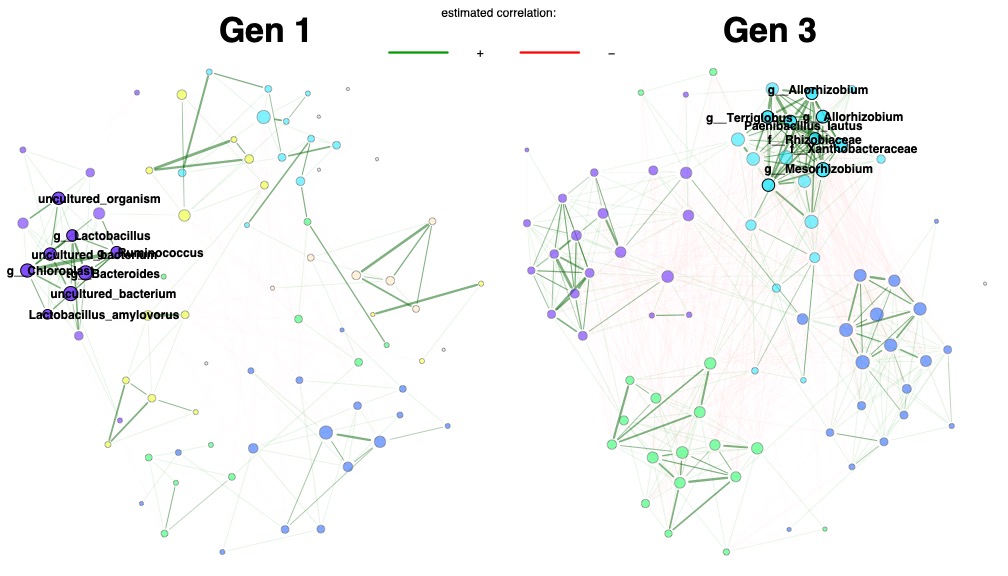

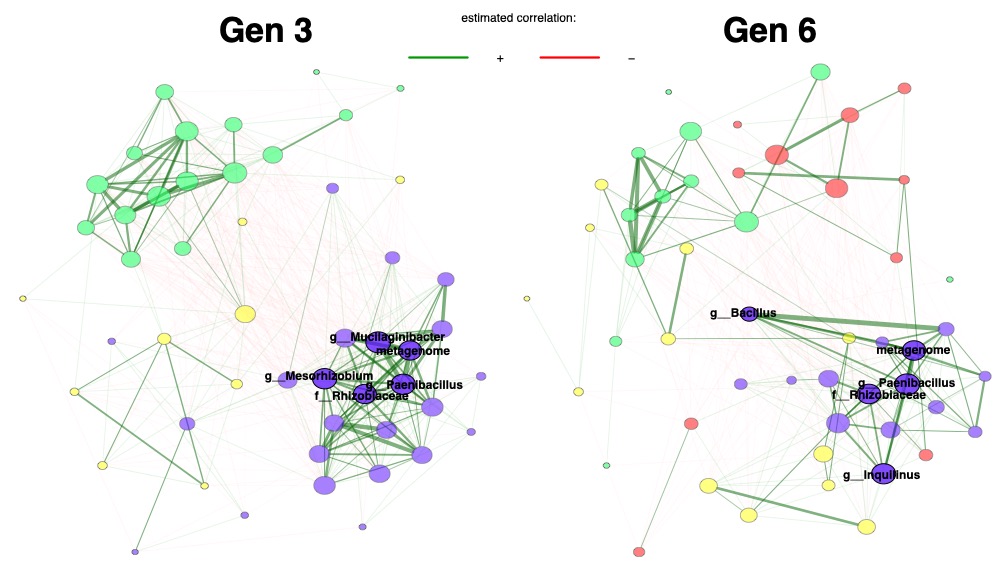


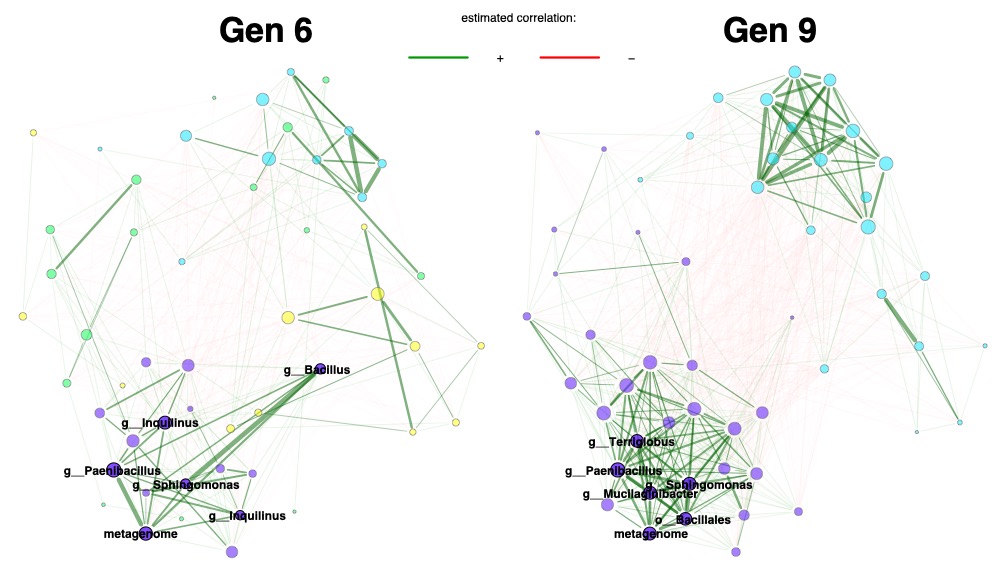


Supplementary Figure S9: Comparison of bacterial associations between samples from different generations. Pearson correlation (>0.3) is used as an association measure, and t-test (< 0.05) is used as a sparse matrix generation method. Eigenvector centrality (>0.90) is used for defining hubs and scaling node sizes. Node colors represent clusters, which are determined using greedy modularity optimization. Clusters have the same color in both networks if they share at least two taxa. Green edges correspond to positive estimated associations and red edges to negative ones. Hubs are highlighted with bold boundaries and corresponding taxon names.
